# Understanding the interplay between CpG island-associated gene promoters and H3K4 methylation^☆^

**DOI:** 10.1016/j.bbagrm.2020.194567

**Published:** 2020-08

**Authors:** Amy L. Hughes, Jessica R. Kelley, Robert J. Klose

**Affiliations:** Department of Biochemistry, University of Oxford, South Parks Road, Oxford OX1 3QU, United Kingdom

**Keywords:** Chromatin, Transcription, DNA methylation, CpG islands, H3K4me3

## Abstract

The precise regulation of gene transcription is required to establish and maintain cell type-specific gene expression programs during multicellular development. In addition to transcription factors, chromatin, and its chemical modification, play a central role in regulating gene expression. In vertebrates, DNA is pervasively methylated at CG dinucleotides, a modification that is repressive to transcription. However, approximately 70% of vertebrate gene promoters are associated with DNA elements called CpG islands (CGIs) that are refractory to DNA methylation. CGIs integrate the activity of a range of chromatin-regulating factors that can post-translationally modify histones and modulate gene expression. This is exemplified by the trimethylation of histone H3 at lysine 4 (H3K4me3), which is enriched at CGI-associated gene promoters and correlates with transcriptional activity. Through studying H3K4me3 at CGIs it has become clear that CGIs shape the distribution of H3K4me3 and, in turn, H3K4me3 influences the chromatin landscape at CGIs. Here we will discuss our understanding of the emerging relationship between CGIs, H3K4me3, and gene expression.

## Highlights

• The deposition of H3K4me3 is intrinsically linked to CpG islands *via* ZF-CxxC domain-containing proteins.
• H3K4me3 levels at CpG islands are regulated by transcription and other chromatin features.
• H3K4me3 influences chromatin architecture at CpG islands and has been proposed to regulate gene expression.
• H3K4me3 may contribute to bistable chromatin states at CpG islands.

## 1 Introduction

The precise control of gene transcription is required for cellular homeostasis and to establish the cell type-specific gene expression patterns that are necessary for complex multicellular development. This regulation primarily relies on site-specific DNA-binding transcription factors (TFs), which shape how the transcriptional machinery engages with gene promoters to initiate transcription. However, in eukaryotes, the chromatin template on which transcription occurs can also profoundly affect how gene expression is established and maintained. For example, the intimate association between DNA and histone octamers within nucleosomes can occlude the binding of TFs and RNA Polymerase II (RNAPII) [1] (reviewed in Li et al., Levine et al. and Venkatesh and Workman [[2], [3], [4]]). Therefore, the position of nucleosomes at gene promoters and regulatory elements can modulate transcriptional output. Furthermore, histone proteins can undergo extensive post-translational modification, including methylation, acetylation, phosphorylation, and ubiquitylation. While these modifications occur on residues throughout histone proteins, the N- or C-terminal tails that extend from the nucleosome core tend to be the most heavily modified and are thought to be particularly important in gene regulation (reviewed in Rothbart et al. and Zhang et al. [5,6]). A subset of histone modifications appears to directly regulate chromatin structure by influencing histone-DNA or nucleosome-nucleosome interactions. Alternatively, histone modifications can create a binding site for, or exclude the binding of, ‘reader’ proteins that in turn nucleate additional activities that influence gene expression.

**Fig. 1:**
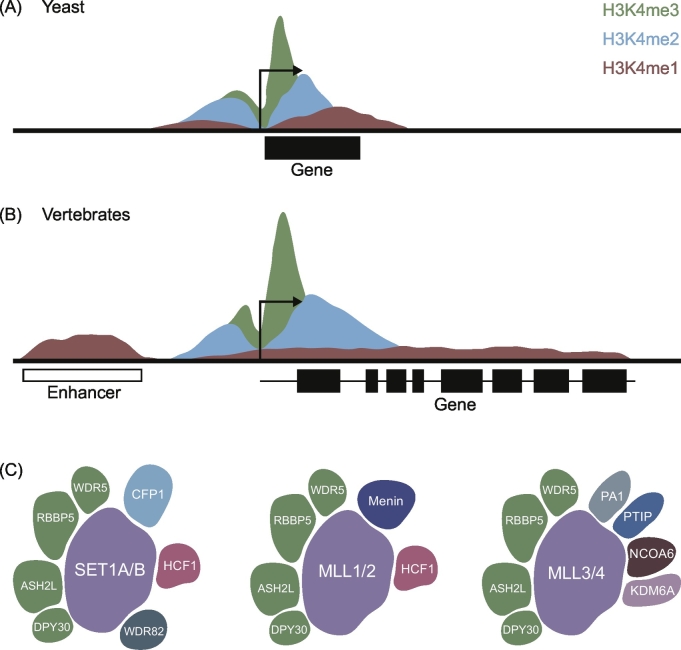
Histone H3 lysine 4 methylation and the H3K4 methyltransferase complexes. (A) and (B) Schematic depictions of the distribution of H3K4 methylation across (A) yeast and (B) vertebrate genes. Arrows indicate transcription start sites. Gene bodies are shown with black boxes representing exons. (C) The subunit composition of mammalian H3K4 histone methyltransferases. The H3K4 HMTs are split into three groups based on the homology of their catalytic subunit: SET1A/B, MLL1/2 and MLL3/4. Shared subunits are depicted in the same colour and position.

Adapted from [266].

One of the most extensively studied histone modifications is the methylation of histone H3 on lysine 4 (H3K4). H3K4 can be mono-, di- or tri-methylated (H3K4me1/2/3). In simple eukaryotes, such as budding yeast, there is a gradient of H3K4 methylation across genes: H3K4me3 is found at gene promoters, H3K4me2 within gene bodies, and H3K4me1 predominates towards the 3′ end of genes (Fig. 1A) [7]. Genome-wide analysis of H3K4 methylation in multicellular eukaryotes, which have larger and more complex genomes, has identified a more distinct pattern of H3K4 methylation. While the enrichment of H3K4me3 at gene promoters is conserved across all eukaryotes, in vertebrates H3K4me1 is more broadly distributed and enriched at enhancers, while H3K4me2 tends to be elevated in regions flanking peaks of H3K4me3 (Fig. 1B) [[8], [9], [10], [11]]. In line with these more diverse patterns of H3K4 methylation, in higher eukaryotes there is an expanded repertoire of enzymes that deposit these modifications. In yeast, a single histone methyltransferase (HMT) complex, known as SET1C, is responsible for placing all H3K4 methylation [12,13]. *Drosophila* have three H3K4 HMT complexes: the TRR complex deposits H3K4me1, while the dSET1 and TRX complexes place H3K4me2/3 at distinct genomic loci [14]. This system has further expanded in vertebrates to produce six H3K4 HMTs [[15], [16], [17], [18], [19], [20]]. Analogous to the functional organisation of the H3K4 HMTs in *Drosophila*, mammalian MLL3/4 complexes are typically associated with H3K4me1 deposition at enhancers, while SET1A/B and MLL1/2 complexes define H3K4me2/3 at gene promoters [[21], [22], [23], [24], [25], [26], [27]]. These H3K4 HMT complexes share a subset of accessory factors but are distinguished based on the identity of their catalytic subunit and are further functionally specialised by the incorporation of complex-specific factors [28] (Fig. 1C and reviewed in Shilatifard [14]).

H3K4me3 is universally associated with gene promoters and has been proposed to play an important role in gene regulation by recruiting additional chromatin modifying enzymes and the transcriptional machinery, and excluding activities typically associated with transcriptional repression (reviewed in Kusch and Hyun et al. [29,30]). Therefore, significant efforts have been made to understand the mechanisms regulating its deposition and function at gene promoters. In budding yeast, H3K4 methylation patterns are predominantly defined by the recruitment of SET1C to sites of active transcription *via* RNAPII [31,32]. However, the diversification of H3K4 methyltransferases in higher eukaryotes has been accompanied by increasingly complex mechanisms for chromatin binding and target site recognition [33]. Transcription alone is insufficient to explain how the SET1A/B and MLL1/2 complexes associate with vertebrate gene promoters. Emerging evidence has revealed an intimate relationship between the SET1A/B and MLL1/2 complexes and a class of vertebrate-specific gene regulatory elements called CpG islands (CGIs). Here we will discuss the relationship between CGIs and H3K4me3, touching on how CGIs shape the patterns of H3K4me3 in vertebrates and how H3K4me3 itself can affect the chromatin architecture around CGI-associated gene promoters to influence gene expression. Since MLL3/4 have been primarily implicated in placing H3K4me1 at enhancers and are not enriched at CGIs, they are beyond the scope of this review. However, their function has been covered extensively elsewhere [34,35].

## 2 CpG islands and H3K4me3

### 2.1 CpG islands delineate H3K4me3 in vertebrate genomes

**Fig. 2:**
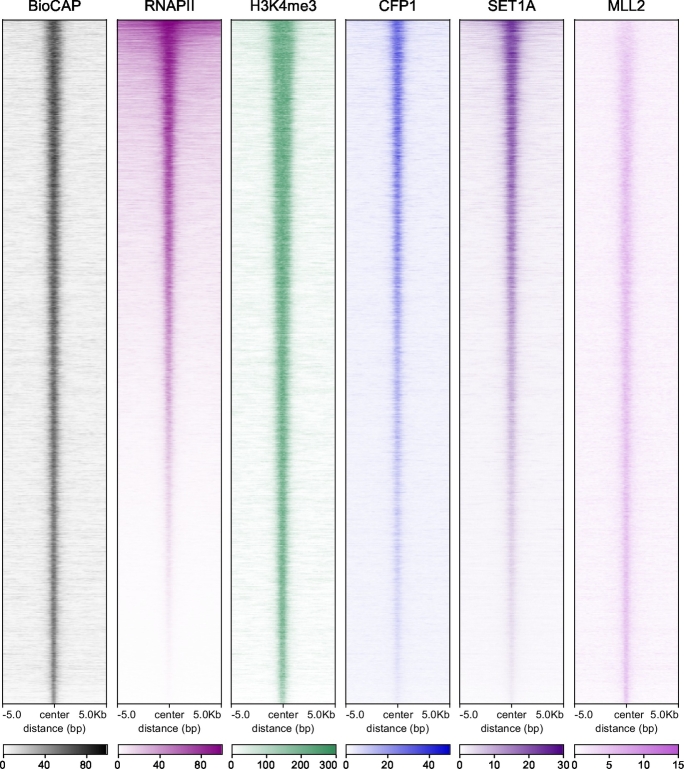
CpG islands and H3K4me3. Heatmaps illustrating non-methylated DNA (BioCAP) [47], RNAPII (transcription) [267], H3K4me3, CFP1, SET1A [61] and MLL2 [25] ChIP-seq signal across all CpG islands in mouse embryonic stem cells. The heatmaps are ranked based on RNAPII signal. H3K4me3 occurs broadly across CpG islands genome-wide. MLL2 associates with the majority of CGIs, whereas CFP1 and SET1A are enriched at CpG islands that are bound by RNAPII.

Much like histones, DNA can be modified in several ways, most notably by the methylation of individual nucleotide bases [5,36,37]. The mammalian genome is pervasively methylated at the 5-position of cytosine bases in the context of CG dinucleotides, which is important for silencing parasitic DNA elements, regulating imprinted gene expression, and supporting X chromosome-inactivation [38,39] (reviewed in Smith and Meissner and Greenberg and Bourc'his [40,41]). Cytosine methylation is also mutagenic, resulting in a global depletion of CG dinucleotides from the mammalian genome [42,43]. However, short (~1–2 kb) regions of the genome, termed CpG islands, remain non-methylated and are enriched for CG dinucleotides [38,44,45]. Approximately 70% of vertebrate gene promoters are associated with CGIs, making them the most common class of promoter in vertebrates [46,47]. CGIs remain free of DNA methylation regardless of the transcriptional state of the associated gene (Fig. 2), suggesting that non-methylated DNA itself does not instruct transcription. However, it has become apparent that CGIs are associated with, and appear to delineate the profiles of, several histone modifications known to have roles in gene regulation. This is exemplified by H3K4me3 which, in vertebrate genomes, correlates most closely with the underlying boundaries of promoter-associated CGIs [47,48]. This is in contrast to simple eukaryotes where H3K4me3 is distributed in a punctate fashion around the transcription start site of actively transcribed genes [7]. Furthermore, although its levels generally scale with transcription, H3K4me3 is present at the majority of CGIs irrespective of whether the associated gene is actively transcribed (Fig. 2). This suggests that the targeting and deposition of H3K4me3 in vertebrates is intrinsically linked to CGIs and not solely reliant on the transcriptional machinery. In support of this possibility, when exogenous CG-rich non-methylated DNA lacking a gene promoter is inserted into the mouse genome, it can acquire H3K4me3 in the absence of RNAPII binding [[49], [50], [51]].

With the exception of CGIs, the majority of the vertebrate genome is methylated in almost all somatic cell types. However, there are specific instances where the mammalian genome becomes markedly hypomethylated, for example in oocytes and at early developmental stages [40,52,53]. If recognition of non-methylated CpG DNA is important for H3K4me3 deposition, one might predict that regions of the genome that lose DNA methylation would acquire H3K4me3. Indeed, Chromatin Immunoprecipitation (ChIP)-sequencing for H3K4me3 in mouse oocytes and during early stages of zygotic development revealed the presence of large domains of H3K4me3 corresponding to non-methylated regions of the genome that are distinct from classical CGIs [54,55]. In oocytes, the appearance of these non-canonical domains of H3K4me3 is dependent on MLL2 [56,57]. However, it should be noted that these large H3K4me3 domains are likely to be cell type-specific or rely on the expression level of specific H3K4 methyltransferases, as removal of DNA methylation in embryonic stem cells (ESCs) does not cause significant spreading of H3K4me3 into non-CGI regions that become hypomethylated [48]. Overall, these observations suggest that the presence of non-methylated CpG DNA, particularly at CG-rich regions of the genome, is essential for guiding the activity of H3K4 HMTs and promoting H3K4me3 deposition.

### 2.2 SET1A/B and MLL1/2 associate with CpG islands *via* ZF-CxxC domains

An important step towards understanding the relationship between CGIs and H3K4me3 came with the discovery that non-methylated CpG dinucleotides are specifically recognised by proteins containing Zinc Finger (ZF)-CxxC domains [58]. The founding member of the ZF-CxxC domain-containing protein family is CFP1 (CXXC1), an essential component of the SET1A/B H3K4 HMT complexes [15,16,59,60]. Genome-wide studies mapping the location of CFP1 and SET1A *in vivo* have indicated that both proteins are enriched at CGI-associated gene promoters (Fig. 2) and *in vitro* binding studies have shown that the ZF-CxxC domain of CFP1 associates specifically with non-methylated CpG dinucleotides [25,[61], [62], [63]]. The DNA binding activity of CFP1 is essential for the association of SET1A with chromatin and normal levels of H3K4me3 at CGIs [61]. This suggests that the SET1A/B complexes are targeted to CGI-associated gene promoters *via* recognition of non-methylated CpG DNA by the ZF-CxxC domain of CFP1. This targeting logic appears to have evolved and been maintained only in the genomes of species that have pervasive DNA methylation and CGIs; CFP1 orthologues in species such as *Drosophila*, which have lost DNA methylation and classical CGIs, do not encode a functional ZF-CxxC domain (reviewed in Krauss and Reuter and Iyer et al. [64,65]).

The MLL1 and MLL2 proteins also contain ZF-CxxC domains that bind specifically to non-methylated CpG dinucleotides *in vitro* [[66], [67], [68], [69], [70]]. Like CFP1, MLL1 and MLL2 also require their ZF-CxxC domains to associate with CGIs *in vivo* [71,72]. Further evidence that the ZF-CxxC domains of MLL proteins are important for targeting to CGIs comes from the study of MLL1 in the context of leukaemia. In such instances, chromosomal translocations generate fusions between the MLL1 N-terminus, which contains its ZF-CxxC domain, and a variety of other proteins, including the transcriptional regulators AF9 and ENL [[73], [74], [75], [76], [77]]. A universal feature of these fusion proteins is the retention of the MLL1 ZF-CxxC domain, which aberrantly targets its fusion partner to CGIs, causing widespread transcriptional defects [70,73,74,[78], [79], [80], [81], [82]]. Loss or mutation of the ZF-CxxC domain in MLL-AF9 and -ENL fusion proteins reduces their capacity for inducing leukaemic cellular transformation, suggesting that the ZF-CxxC domain is crucial for their association with chromatin [69,71,78,79].

Although the presence of non-methylated DNA appears to be sufficient to create H3K4me3-modified chromatin and the ZF-CxxC domains inherent to the SET1A/B and MLL1/2 complexes can target them to CGIs, this single interaction does not completely explain the localisation and activity of these complexes. Whilst MLL2 binds broadly to CGIs *in vivo* (Fig. 2), it only appears to contribute significantly to H3K4me3 at a subset of lowly transcribed CGI-associated gene promoters [24,25]. In contrast, SET1A/B complexes are preferentially enriched at actively transcribed CGI-associated gene promoters (Fig. 2) [25,61,83]. Furthermore, *in vitro* measurements suggest that the affinity of ZF-CxxC domains for non-methylated CpG dinucleotides is relatively low (~0.1–7 μM) and is therefore unlikely to constitute the sole chromatin binding determinant for these complexes [63,67,68]. Therefore, significant efforts have been made to understand the additional mechanisms by which H3K4 HMT complexes recognise their CGI-associated target sites to deposit H3K4me3.

### 2.3 Multivalent chromatin interactions restrict H3K4 HMTs to a subset of CpG island promoters

Many chromatin-associated proteins exploit multivalent interactions that rely on a series of low-affinity binding modules to provide specificity and elevated affinity for their target sites [84]. In addition to binding non-methylated CpG DNA *via* its ZF-CxxC domain, CFP1 also recognises H3K4me3 through its Plant HomeoDomain (PHD domain) [61,85,86]. This provides the SET1A/B complexes with a preferential affinity for CGIs that are already modified by H3K4me3. Interestingly, mutations that disrupt H3K4me3 binding by CFP1 do not completely eliminate CFP1 occupancy at CGIs. In fact, in the absence of a functional PHD domain, residual CFP1 binding is uniformly distributed across CGIs and correlates with CpG content. In contrast, if the ZF-CxxC domain of CFP1 is mutated there remains little, if any, CFP1 occupancy at CGIs [61]. This suggests that the ZF-CxxC domain of CFP1 is the primary determinant of its affinity for chromatin. Multivalent engagement *via* the PHD domain then stabilises binding to provide additional specificity that enriches CFP1 and SET1A/B complexes at CGIs with pre-existing H3K4me3.

MLL1 and MLL2 also contain multiple PHD domains, some of which bind to H3K4me3 [71,[86], [87], [88], [89], [90]]. The H3K4me3-binding PHD domain of MLL1 can contribute to its occupancy at some target sites, however the relevance of these PHD domains for MLL1/2 complex localisation genome-wide has not been defined [71,88]. Nevertheless, the genome-wide occupancy of MLL2 appears to be more uniformly distributed across CGIs than CFP1 and SET1A (Fig. 2) [25]. This suggests that if the PHD domains of MLL1 and MLL2 do associate with H3K4me3 *in vivo*, they do not enrich MLL1/2 complexes at the subset of CGIs that have elevated H3K4me3.

## 3 Regulating H3K4me3 at CpG island-associated gene promoters

### 3.1 Is H3K4me3 deposition directed by active transcription?

**Fig. 3:**
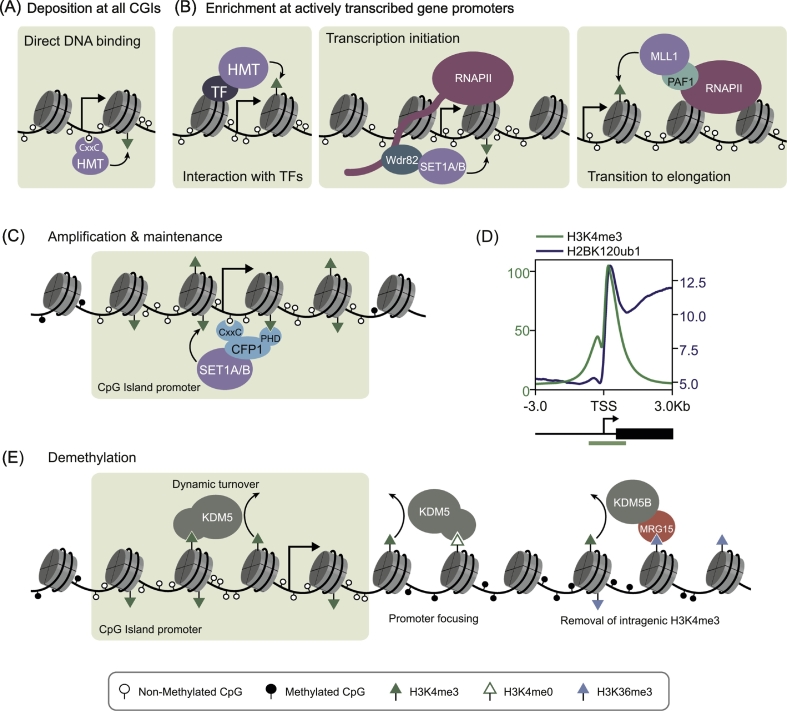
Mechanisms that regulate H3K4me3 at CpG islands. (A) H3K4me3 HMT complexes can deposit H3K4me3 across all CpG islands by binding directly to non-methylated CpG dinucleotides *via* their ZF-CxxC domains. The position of the transcription start site is illustrated as an arrow and a legend for the chromatin modifications is shown at the bottom of this figure. (B) Mechanisms to enrich H3K4me3 at actively transcribed gene promoters. These include interaction of HMT complexes with transcription factors (TFs) or interactions with the transcription machinery during initiation and elongation. (C) A mechanism to amplify and maintain H3K4me3 once initiated. CFP1 stabilises SET1A/B complexes at actively transcribed CGI-associated gene promoters through multivalent interactions with non-methylated CpG dinucleotides and pre-existing H3K4me3. (D) A metaplot illustrating the distribution of H3K4me3 and H2BK120ub1 ChIP-seq signal at CGI associated gene promoters in mouse embryonic fibroblasts [105]. H3K4me3 is distributed around the TSS and over the CGI, whereas H2BK120ub1 peaks downstream of the TSS and is also enriched throughout the gene body. The schematic below indicates the position of the TSS as an arrow, CGI promoter as a green box and gene body as a black box. (E) Mechanisms through which demethylation by KDM5 proteins could shape H3K4me3. These include dynamic turnover of H3K4me3, focussing of H3K4me3 at gene promoters, and removal of spuriously deposited H3K4me3 in gene bodies.

Unlike the MLL1/2 complexes, which broadly occupy all CGI promoters, the SET1A/B complexes are enriched at actively transcribed gene promoters and are proposed to contribute centrally to H3K4me3 at these loci [26,61,[91], [92], [93], [94]]. Interestingly, the selectivity of the SET1A/B complexes for actively transcribed gene promoters appears to rely on multivalent interactions with CGIs and pre-existing H3K4me3 [61]. How, therefore, is H3K4me3 enriched at actively transcribed CGI-associated gene promoters to support preferential binding of the SET1A/B complexes and further elevation of H3K4me3? Whilst all four H3K4me3 HMTs can bind to CGIs *via* their ZF-CxxC domains (Fig. 3A) and the presence of CpG island DNA is sufficient for H3K4me3 deposition *in vivo*, this DNA-based recognition modality alone cannot distinguish between transcribed and non-transcribed genes [[49], [50], [51]]. Additional mechanisms must therefore exist to enrich H3K4me3 specifically at actively transcribed gene promoters. One possible explanation may be that the H3K4 HMTs are specifically targeted to gene promoters during the process of gene activation. If so, deposition of H3K4me3 through transcription-coupled mechanisms could then support multivalent recognition of non-methylated CpG DNA and H3K4me3 *via* CFP1-containing SET1A/B complexes.

In support of this hypothesis, the SET1A/B and MLL1/2 complexes interact with a number of sequence-specific DNA binding transcription factors and long non-coding RNAs (Fig. 3B) (reviewed in Bochyńska et al. and Crump and Milne [33,34]), although the relevance of these interactions for H3K4me3 deposition genome-wide have not been fully dissected. Additionally, the WDR82 subunit of the SET1A/B complexes is proposed to directly interact with the Serine 5-phosphorylated C-terminal heptad repeat domain of initiating RNAPII [95,96]. This interaction could elevate H3K4me3 at gene promoters during transcription initiation, either by stabilising SET1A/B binding, or by stimulating their catalytic activity (Fig. 3B). In agreement with this possibility, there is a widespread requirement for WDR82 in the acquisition of H3K4me3 during lipopolysaccharide-induced gene induction in macrophages [94]. However, the removal of WDR82 does not result in a global loss of H3K4me3 from CGI-associated gene promoters in the steady state, suggesting that WDR82 is not required for maintaining H3K4me3 at CGI promoters [94].

In addition to the initiation of gene transcription, the transition from initiation to elongation has also been implicated in the deposition of H3K4me3. MLL1 directly interacts with PAF1 (Polymerase Associated Factor 1), a core component of the PAF1 complex which facilitates transcription elongation [71,97,98]. Transient recruitment of MLL1 during the transition from initiation to elongation could lead to H3K4me3 deposition at actively transcribed genes (Fig. 3B). However, immunofluorescence experiments have shown that there is a minimal overlap in the localisation of MLL1 and PAF1, suggesting that this interaction may only occur at a small number of loci *in vivo* [71]. Additionally, it seems unlikely that MLL1/2 are responsible for elevated H3K4me3 at actively transcribed genes given that they broadly occupy CGI-associated gene promoters regardless of their transcriptional state [25,72]. While the precise mechanisms governing H3K4me3 enrichment at actively transcribed CGI-associated gene promoters remain poorly defined, it seems likely that the process of active transcription is required. Once H3K4me3 is elevated at actively transcribed gene promoters, this could initiate a simple feedback mechanism whereby the SET1A/B complexes are stabilised at transcribed genes *via* multivalent binding to non-methylated CpG DNA and H3K4me3, further amplifying H3K4me3 and SET1A/B complex occupancy (Fig. 3C).

### 3.2 Regulating the activity of the H3K4 HMTs at CpG island-associated gene promoters

Whilst CGIs play a central role in specifying how the H3K4 HMTs identify target sites in the genome, it is also clear that other features of CGI chromatin can significantly affect H3K4me3 deposition. For example, mono-ubiquitylation of histone H2B on lysine 120 (H2BK120ub1) stimulates the enzymatic activity of both MLL1 and SET1A on mononucleosomal substrates *in vitro*, and H2BK120ub1 is a prerequisite for H3K4me3 deposition on reconstituted polynucleosomal chromatin templates [[99], [100], [101], [102]]. Recent cryo-EM structures of H3K4 methyltransferases bound to ubiquitylated nucleosomes have revealed possible mechanisms for this regulation. Structures of the yeast SET1C complex bound to both unmodified and H2BK120ub1 nucleosomes revealed that a conserved arginine-rich motif in the N-SET domain of SET1C binds to H2BK120ub1 and induces a conformational change which may activate HMT activity [103,104]. Although this motif is conserved in vertebrate SET1A/B, it is not retained in MLL proteins. Instead, studies of an MLL1 complex bound to unmodified and H2BK120ub1 nucleosomes suggested that activation by H2BK120ub1 may arise from restriction of MLL1 binding geometry and enhanced affinity for nucleosome substrates [100].

H3K4 HMT activity is clearly regulated by H2BK120ub1 *in vitro*, however the relevance of this for H3K4me3 deposition *in vivo* remains poorly defined. Like H3K4me3, the level of H2BK120ub1 within genes correlates with gene activity [105,106]. However, the distribution of H2BK120ub1 within genes differs significantly from that of H3K4me3. Whilst H3K4me3 is present at gene promoters and over CGI elements, H2BK120ub1 is distributed throughout the transcribed regions of genes with only modest enrichment towards the 5′ end [105,106] (Fig. 3D). The mechanism by which H2BK120ub1 might contribute to the formation of H3K4me3 domains *in vivo* therefore remains unclear*. In vitro* observations suggest H2BK120ub1 may simply be a prerequisite for H3K4me3 deposition, and depletion of H2BK120ub1 has been associated with reduced H3K4me3 *in vivo* [101,102,105]. However, H3K4me3 seems to occur independently of H2BK120ub1 in some differentiated cell types [107]. It is also possible that H2BK120ub1 shapes the level or distribution of H3K4me3. Indeed, loss of H2BK120ub1 following knockout of the H2B E3 ligase component RNF40 is associated with reduced H3K4me3 predominantly on the genic side of actively transcribed gene promoters [105].

Whilst H2BK120ub1 promotes H3K4 methylation, other histone modifications can prevent H3K4me3 deposition. An inverse relationship between asymmetrically dimethylated histone H3 arginine 2 (H3R2me2a) and H3K4me3 has been observed in several instances; increased H3R2me2a following overexpression of the methyltransferase PRMT6 is associated with a decrease in bulk H3K4me3, whilst knockout of PRMT6 leads to reduced H3R2me2a and elevated H3K4me3 at gene promoters [108,109]. Interestingly, occupancy of MLL1 and the H3K4 HMT subunit WDR5 are also inversely correlated with H3R2me2a, suggesting that H3R2me2a may prevent H3K4 HMT binding [[109], [110], [111]]. Given the close proximity of H3K4 and H3R2 in the histone H3 tail, many H3K4me3-binding proteins make extensive contacts with H3R2 and its methylation has been shown to alter their binding affinity in a number of instances [[112], [113], [114], [115], [116], [117]]. Notably, H3R2me2a could affect binding of the SET1A/B complexes since the PHD domain of CFP1 has a lower affinity for doubly modified H3R2me2aK4me3 peptides than for singly modified H3K4me3 peptides *in vitro* [113]. Furthermore, MLL/SET1 complexes are less active towards H3 peptides already carrying R2me2a, suggesting that H3R2me2a may prevent H3K4me3 deposition by limiting both the binding and activity of H3K4 HMTs [110,111]. The functional relevance of the crosstalk between H2R2me2a and H3K4me3 is not clear; H3R2me2a has generally been associated with transcription repression, however H3R2me2a colocalises with H3K4me3 at actively transcribed gene promoters [108,109,111]. It has been proposed in this context that H3R2me2a functions to constrain or fine-tune H3K4me3 levels and transcriptional activity, however further work is required to explore this possibility.

### 3.3 Shaping H3K4me3 after its deposition

The observed distribution of most histone modifications is dictated by mechanisms that regulate both deposition and removal. In contrast to some other trimethyl lysine modifications, which are stable over several cell divisions, H3K4me3 is relatively dynamic, with a half-life of 7 h [118]. In agreement with the dynamic nature of H3K4me3, there are four well-characterised H3K4me3 demethylases in mammals: KDM5A-D [[119], [120], [121], [122], [123]]. While these enzymes have been implicated in developmental processes and are perturbed in a range of cancers, there remains a relatively limited understanding of how they function to regulate H3K4 methylation genome-wide [[124], [125], [126]]. All four KDM5 family members contain a DNA-binding ARID domain and multiple PHD domains that may be responsible for chromatin binding [125,127,128]. The third PHD domain of KDM5A binds H3K4me3, which may allow for direct recruitment of H3K4 demethylase activity to substrate chromatin [129]. *In vivo*, KDM5B binds to sites in the genome that are enriched for H3K4me3, implicating it in the dynamic turnover of H3K4me3 at these loci (Fig. 3E, left) [130]. KDM5 proteins also appear to act near gene promoters to prevent H3K4me3 spreading into gene bodies, which may serve to focus H3K4me3 specifically at CGI promoters (Fig. 3E, centre) [130]. In addition, KDM5B has been shown to associate with the H3K36me3-binding protein MRG15, which may target it to intragenic regions to remove aberrantly deposited H3K4me3 (Fig. 3E, right) [131]. Interestingly, the PHD1 domains of KDM5A and KDM5B have been shown to allosterically activate these enzymes upon binding their own product, H3K4me0 [[132], [133], [134]]. This positive feedback mechanism may drive propagation of demethylation along nucleosomes to remove large H3K4me3 domains upon switching to a transcriptionally inactive state, or serve to maintain methylation-free chromatin domains [131,135]. Demethylases could also contribute to the enrichment of H3K4me3 at actively transcribed promoters by counteracting H3K4me3 accumulation at lowly transcribed loci. Our understanding of how demethylases shape H3K4me3 is still developing and further work is required to define their role at CGI-associated gene promoters.

## 4 How does H3K4me3 influence CpG island chromatin and gene expression?

### 4.1 CpG islands, H3K4me3, and DNA methylation

A key feature of CGI-associated gene promoters is that they tend to remain free of DNA methylation [38,41]. Given that the abnormal methylation of CGI gene promoters is associated with stable transcriptional repression, for example in cancer, it has been proposed that the non-methylated state of CGIs contributes to the maintenance of a chromatin environment that is responsive to gene regulatory cues [[136], [137], [138], [139]]. However, it is unknown how CGIs remain refractory to DNA methylation when the surrounding genome is pervasively methylated. Because DNA methylation and H3K4me3 are almost entirely mutually exclusive, it was proposed that H3K4 methylation may prevent the methylation of CGIs [52,[140], [141], [142], [143]]. Indeed, a direct inverse relationship between H3K4me3 and DNA methylation has now been observed in a number of studies [48,53,144,145]. In mice, perturbation of H3K4me3 is associated with increased DNA methylation at certain genomic loci, while the expression of a mammalian DNA methyltransferase in yeast, an organism devoid of DNA methylation, results in the accumulation of aberrant DNA methylation at H3K4me3-depleted regions [18,141,144,[146], [147], [148]]. This atypical accumulation of DNA methylation in yeast is further enhanced in the absence of H3K4me3, suggesting that DNA methyltransferase activity is blocked at sites marked by H3K4me3 [141,148].

A family of DNA methyltransferases (DNMTs) catalyses the methylation of CG dinucleotides throughout the mammalian genome. DNMT3A/B, aided during early development by their non-catalytic cofactor DNMT3L, primarily catalyse *de novo* DNA methylation. DNMT1 is a maintenance methyltransferase which ensures that DNA methylation patterns are re-established after cell division (reviewed in Gowher and Jeltsch [149]). Detailed structural and biochemical studies of the DNMTs have uncovered potential mechanisms by which H3K4me3 could counteract the deposition of DNA methylation at CGIs. The ADD domains of DNMT3A/B and DNMT3L preferentially bind to the N-terminal tail of histone H3 when H3K4 is non-methylated [[150], [151], [152], [153]]. Rendering the DNMT3A ADD domain insensitive to the H3K4 methylation state in mouse ESCs results in increased DNMT3A binding and CGI-associated DNA methylation at sites with H3K4me3 [153]. This suggests that H3K4 methylation precludes association of the *de novo* DNA methyltransferases with CGIs and therefore prevents the acquisition of DNA methylation. However, the relevance of the ADD domains for correct genomic targeting *in vivo* has been debated [154,155].

**Fig. 4:**
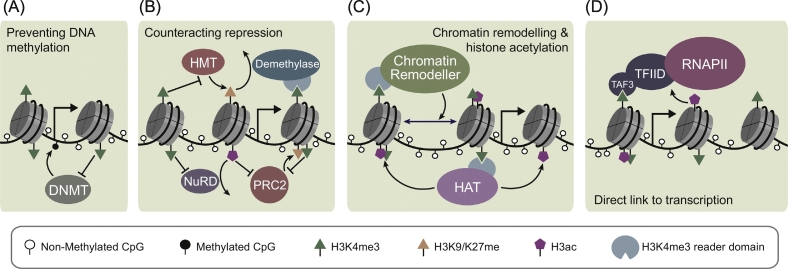
The relationship between H3K4me3 and transcriptionally permissive chromatin. (A) H3K4me3 has been proposed to counteract methylation of CpG islands by preventing both binding and activation of the *de novo* DNA methyltransferases (DNMTs). The position of the transcription start site is illustrated as an arrow in each panel and a legend for the chromatin modifications is shown below the panels. (B) H3K4me3 counteracts acquisition of repressive chromatin modifications by preventing binding of histone methyltransferases (HMTs) while promoting removal of repressive methylation by stabilising binding of demethylases. In addition to inhibiting its catalytic activity, H3K4me3 may also inhibit PRC2 by preventing binding of NuRD and hence indirectly reduce PRC2 association with chromatin. (C) Histone acetyltransferases (HATs) and chromatin remodellers bind to H3K4me3 and contribute to an accessible and transcriptionally permissive chromatin architecture at CGI-associated gene promoters. (D) The TAF3 subunit of TFIID binds H3K4me3 and promotes pre-initiation complex formation, providing a direct link to transcription. This interaction is enhanced by histone acetylation.

It has also been proposed that binding to H3 regulates the enzymatic activity of DNMT3A, which is thought to reside in an autoinhibited state when not bound to chromatin. Release of this autoinhibition is dependent on DNMT3A association with H3K4me0 [152,156,157]. This could provide an additional layer of protection against DNA methylation at CGIs, since aberrant binding of DNMT3A to H3K4me3-containing chromatin would fail to release its autoinhibition. By preventing DNA methylation at CGIs (Fig. 4A), the presence of H3K4me3 could ensure that they are maintained in a transcriptionally permissive state.

### 4.2 Does H3K4me3 counteract transcriptional repression?

ZF-CxxC domain-containing proteins are present in protein complexes associated with both transcriptional activation and repression. It is therefore essential that the CGI-associated gene promoters of actively transcribed genes are resistant to the activities of repressive complexes that can bind to these loci. In agreement with this, there is evidence that H3K4me3 may counteract chromatin states associated with transcriptional repression. For example, H3K4me3 has been implicated in inhibiting the deposition of repressive, heterochromatin-associated histone modifications, most notably methylation of H3K9 and H3K27. H3K4me3 has been proposed to regulate H3K9 methylation by inhibiting binding of the H3K9 HMTs, preventing its deposition, and also by stabilising binding of H3K9 demethylases, thereby promoting its removal from gene promoters (Fig. 4B) [[158], [159], [160], [161], [162], [163], [164], [165], [166], [167], [168], [169], [170], [171], [172], [173]].

The relationship between H3K4me3 and H3K27me3, a histone modification catalysed by the Polycomb repressive system, has also been extensively studied (Reviewed in Voigt et al. [174]). From *in vitro* experiments, it is clear that H3K4me3 inhibits H3K27me3 deposition by Polycomb-repressive complex 2 (PRC2) on the same N-terminal tail of histone H3. However, the mechanism for this inhibition remains unclear [[175], [176], [177], [178]]. There is also *in vitro* evidence that binding of the NuRD (Nucleosome Remodelling and Deacetylase) complex to histone H3 peptides is reduced in the presence of H3K4me3 [86,172,[179], [180], [181], [182], [183], [184]]. Since NuRD-mediated deacetylation promotes PRC2 engagement with chromatin, this may be a mechanism whereby H3K4me3 indirectly counteracts the formation of transcriptionally repressive Polycomb domains (Fig. 4B) [185]. Somewhat counterintuitively, however, ChIP-sequencing in mammalian cells has shown that the localisation of H3K27me3 and H3K4me3 can extensively overlap at CGIs, particularly in ESCs. This so-called ‘bivalent’ chromatin has been proposed to retain genes in a repressed, but ‘poised’, configuration to enable rapid activation during differentiation [[186], [187], [188]]. There is, however, currently little evidence for the role of bivalent chromatin in supporting gene activation [24,25,72,189].

### 4.3 Regulation of histone acetylation and chromatin remodelling by H3K4me3

Biochemical approaches aimed at identifying factors that interact with the N-terminal tail of histone H3 have identified a number of proteins that specifically bind to, or are excluded by, H3K4me3 [30,86,89,190]. These include several protein complexes associated with histone acetylation and chromatin remodelling.

Much like H3K4me3, histone acetylation is found at active gene promoters [9,191]. However, in contrast to histone methylation, which does not affect the charge of lysine residues, histone acetylation neutralises their positive charge, weakening histone tail-DNA interactions and counteracting chromatin compaction [192]. It is therefore likely that the presence of histone acetylation at CGI-associated gene promoters increases their accessibility and therefore contributes to a transcriptionally permissive chromatin state. The close relationship between H3K4me3 and histone acetylation is exemplified by the SAGA (Spt-Ada-Gcn5 Acetyltransferase) chromatin-modifying complex, which has been implicated in transcriptional activation [193]. The SGF29 (SAGA-associated Factor 29) subunit of the SAGA complex binds to H3K4me3 *via* its tandem Tudor domains [89,194]. This is important for the association of the SAGA complex with chromatin and acetylation of its target loci. However, despite there being a major reduction in histone acetylation upon H3K4me3 perturbation *in vivo*, there is evidence that the relationship between H3K4me3 and histone acetylation may not be so straightforward [195,196]. The mammalian ING proteins (INhibitor of Growth 1–5) each contain a PHD domain that preferentially binds to H3K4me3 [[197], [198], [199], [200], [201], [202], [203], [204]]. While ING3-5 associate with histone acetyltransferase complexes, ING1 and ING2 associate with the SIN3 histone deacetylase complex, which removes histone acetylation and can therefore counteract gene expression [[205], [206], [207], [208], [209]]. It is possible that, by recruiting both histone acetyltransferases and histone deacetylases to CGI-associated gene promoters, H3K4me3 contributes to the dynamics and/or distribution of histone acetylation [[210], [211], [212]]. Further work is required to better define how histone acetylation is regulated at CGI gene promoters, although H3K4me3 appears to be central to this process.

Nucleosome occupancy and phasing at gene promoters have been widely implicated in regulating gene expression (reviewed in Lai and Pugh and Brahma and Henikoff [213,214]). Actively transcribed CGI-associated gene promoters, which have high H3K4me3, are characterised by dynamic nucleosomes in which the underlying DNA is more accessible compared to the CGI-associated promoters of lowly transcribed genes [215,216]. It has been proposed that this chromatin state relies on H3K4me3 and its capacity to stabilise the binding of chromatin remodelling enzymes [217,218]. For example, BPTF (Bromodomain and PHD finger-containing Transcription Factor), a subunit of the NURF (NUcleosome Remodelling Factor) chromatin-remodelling complex, has a PHD domain that binds to H3K4me3 [219,220]. NURF utilises the energy derived from ATP-hydrolysis to slide nucleosomes on DNA, and has therefore been implicated in transcriptional regulation [221]. BPTF association with H3K4me3 is required for appropriate NURF complex binding and remodelling activity on chromatin *in vivo* [217,219]. Interestingly, BPTF also has a bromodomain that binds acetylated histone H4 tails and enables it to engage in multivalent interactions with chromatin carrying both H3K4me3 and H4 acetylation [222]. These modifications at actively transcribed genes may therefore reinforce a dynamic and accessible chromatin state to support ongoing gene transcription. In addition to BPTF/NURF, the ATP-dependent chromatin remodelling enzyme CHD1 (Chromodomain Helicase DNA binding protein 1) also binds to H3K4me3 *via* its double chromodomains [223,224]. H3K4me3 has been proposed to enhance the nucleosome remodelling activity of CHD1, which is thought to contribute to the maintenance of a transcriptionally-permissive chromatin state at gene promoters [225,226]. Together these observations suggest that H3K4me3 and histone acetylation may promote gene expression, at least in part, by recruiting chromatin remodelling enzymes to actively transcribed CGI-associated gene promoters (Fig. 4C).

### 4.4 H3K4me3 and a direct link to the transcriptional machinery

Most of the characterised H3K4me3 reader proteins associate with activities that modify or remodel chromatin, and many of these have proposed roles in gene regulation. However, there is also evidence that H3K4me3 may directly affect the transcriptional machinery itself. The TAF3 subunit of the TFIID general transcription factor complex contains a PHD domain that associates with H3K4me3 and promotes pre-initiation complex (PIC) formation on *in vitro* transcription templates [86,114,115]. In a recent model of RNAPII engagement with chromatin, it was proposed that the association of TFIID with nucleosomes precedes engagement of TBP (TATA-Binding Protein) with DNA [227]. Therefore, additional mechanisms that support nucleosome binding by TFIID, like recognition of H3K4me3, could be more important for initiation of RNAPII-mediated gene transcription than previously appreciated [228]. Indeed, TAF3 binding to chromatin *in vivo* significantly overlaps with H3K4me3 at CGI-associated gene promoters, and binding to H3K4me3 is essential for recruitment of TBP and the appropriate induction of many TAF3 target genes [229,230]. It is therefore possible that, in addition to contributing to a transcriptionally permissive chromatin environment, H3K4me3 may directly affect RNAPII function by mediating PIC formation at CGI-associated gene promoters (Fig. 4D). Interestingly, binding of TFIID to H3K4me3-containing chromatin is also enhanced by histone acetylation, another key feature of actively transcribed CGI-associated gene promoters [114,230] (Section 4.3.).

### 4.5 Does H3K4me3 regulate gene expression?

As we have previously described, H3K4me3 correlates with gene transcription, contributes to a chromatin environment that is seemingly permissive to gene transcription, and has a direct link to the transcriptional machinery (Fig. 2, Fig. 4). It has therefore been proposed that H3K4me3 directly contributes to gene transcription [114,229]. In support of this, it has been shown in some contexts that efficient transcription *in vitro* is dependent on H3K4 methylation, though in others its contribution is minimal [101,225,229,231,232]. However, there remains little evidence directly linking the loss of H3K4me3 to major genome-wide alterations in gene expression *in vivo*. For example, yeast strains completely lacking SET1C and H3K4me3 are viable and have only mild transcriptional defects. This may be because yeast TAF3 lacks the H3K4me3-binding PHD domain which has been proposed to stimulate PIC formation in mammalian cells [13,[233], [234], [235], [236], [237], [238], [239], [240], [241], [242], [243]]. Studying the requirement of H3K4me3 for gene expression in vertebrates is complicated by the fact that there are multiple H3K4me3 HMTs, which may have overlapping function and also methyltransferase-independent functions [83,244]. While depletion of components of the SET1A/B complexes in vertebrate cells results in clear defects in gene expression, these changes are subtle and often manifest as decreases in gene expression at some genes and increases in gene expression at others, and there is limited correlation between gene expression changes and alterations in H3K4me3 [61,94,195,245,246]. This suggests that, at least in the steady state, alterations in H3K4me3 do not have a predictable effect on gene expression [247].

It has been proposed that the acquisition of H3K4me3 may be important for gene induction, for example during differentiation [83,248,249]. However, perturbation of the SET1A/B complexes during LPS-mediated gene induction in macrophages and DNA damage-induced gene expression in ESCs does not result in widespread transcriptional defects, despite reduced H3K4me3 levels at activated genes [94,195]. Similarly, the removal of MLL2 in ESCs reduces H3K4me3 at lowly transcribed or inactive CGI-associated genes but does not affect the induction of the majority of these genes during retinoic acid-induced differentiation, or differentiation into embryoid bodies [24,25,72,189]. Therefore, at least under the kinetic parameters examined thus far, there remains little evidence that H3K4me3 acquisition is essential for gene induction.

Single-cell measurements have shown that the transcription of genes associated with broad domains of H3K4me3 is more consistent, or less varied, between cells [250]. This transcriptional consistency appears to be dependent on the presence of H3K4me3, suggesting that H3K4me3 somehow promotes more consistent transcription. One could imagine that cell-to-cell variation in gene expression could be deleterious to cell fate decisions during differentiation and to coordinated cellular functions in tissues that require homogeneous gene expression. This may explain why SET1A/B and MLL1/2 complexes are essential for normal embryonic development, but their removal in cell culture systems results in seemingly modest effects on gene expression [26,[251], [252], [253], [254]]. It will be important in future work to determine whether the developmental defects that arise following removal of SET1A/B and MLL1/2 are due to alterations in H3K4me3 and transcriptional consistency.

### 4.6 Does H3K4me3 contribute to chromatin bistability at CGI-associated promoters?

**Fig. 5:**
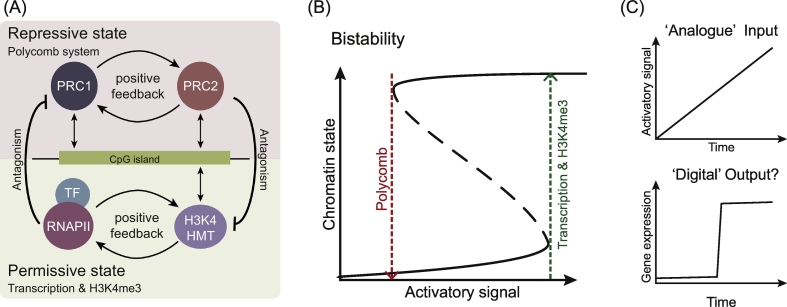
Do CpG island-associated gene promoters and chromatin bistability shape transitions in gene expression? (A) Both the Polycomb system (PRC1 and PRC2) and H3K4 histone methyltransferases (HMTs) can engage with or ‘sample’ CGIs *via* ZF-CxxC domain-containing proteins. Self-reinforcing positive feedback loops and mutual antagonism inherent to these systems could create bistable chromatin states at CGIs. (B) A schematic illustrating the proposed bistable chromatin state at CpG island-associated gene promoters. The transcriptionally repressive Polycomb state is sustained by feedback mechanisms that also antagonise the permissive state (A). We envisage that this Polycomb dependent repressive state constrains transcription until gene activation signals reach a threshold where inhibition is overcome and transcription initiates effectively. This would then result in a transcription-dependent switch to a permissive chromatin state, characterised by H3K4me3 and transcriptional activity (green arrow). This transcriptionally permissive state is maintained through self-reinforcing feedback mechanisms that also antagonise the Polycomb state (A). The permissive state is thereby maintained unless the activatory signal drops below a certain level where feedback can no longer be sustained and transcription does not persist. This would then cause a switch back to the repressive Polycomb chromatin state (red arrow). (C) The interaction between regulatory signals and bistable chromatin states (A,B) at CGI-associated gene promoters could shape gene expression transitions such that graded analogue gene regulatory inputs are translated into switch-like digital gene expression outputs.

Although the precise role of H3K4me3 in vertebrate gene expression has yet to be fully defined, its proposed contribution to transcriptional consistency suggests that this system may regulate gene expression in a manner that is masked by ensemble measurements and may only be evident when assayed with appropriate kinetic measurements or in single-cell experiments. We and others have previously proposed that CGIs may create bistable chromatin states at gene promoters to regulate gene expression [138,174,[255], [256], [257], [258], [259], [260], [261], [262], [263]]. In the context of such a system, we propose that repressive Polycomb chromatin states maintained by feedback mechanisms at inactive genes constrain gene activation signals until an appropriate gene induction threshold is reached. In contrast, following productive initiation of transcription, the establishment of permissive chromatin states potentiates transcription, provides memory that the gene was recently transcribed, and allows for gene expression to be maintained even when levels of activatory signal drop below the threshold required for initial activation (Fig. 5A, B). We envisage that this switching between chromatin states could allow analogue gene induction signals to be converted into digital gene expression outputs through the behaviour of the underlying chromatin at CGIs (Fig. 5C).

In the context of this model for chromatin bistability at CGIs, we envisage that the H3K4 HMT complexes dynamically engage with, or sample, CGIs *via* their ZF-CxxC domains, but this is insufficient for stable occupancy and high level H3K4me3 deposition. During gene activation, transcription factor-dependent or cotranscriptional recruitment of H3K4 HMTs would lead to increased H3K4me3 at target gene CGIs. This elevated H3K4me3 associated with gene activation could be recognised by PHD domains in H3K4 HMT complexes to support multivalent binding that would further elevate H3K4me3 at transcribed CGI-associated gene promoters (Fig. 3). Elevated H3K4me3 would in turn promote gene transcription, creating a feedback loop that could also counteract repressive chromatin states (Fig. 4, Fig. 5A). Given that vertebrate gene expression is inherently stochastic, a permissive H3K4me3 chromatin state at transcribed gene promoters may support uniformity in gene expression and provide chromatin-based memory of recent transcriptional events [264].

Recent mathematical models integrating the known activities of complexes that drive permissive and repressive chromatin states at CGIs have provided some evidence that bistable chromatin states are likely to exist, and that such systems could potentially modulate transcriptional behaviours in a manner that is consistent with the ideas described here and previously [138,174,[255], [256], [257], [258], [259], [260], [261], [262], [263],^265^]. In the context of these emerging models, it is important to highlight that previous work examining the effects of H3K4me3 perturbation on gene induction relied on ensemble transcriptional measures. Such measures are incapable of distinguishing between analogue and digital transcriptional behaviours and lack the time resolution required to capture kinetic contributions to these gene expression transitions. Therefore, future studies will require suitably quantitative and time-resolved methods to examine the contribution of CGIs and H3K4me3 to gene expression and to test whether chromatin bistability may contribute to appropriate gene expression transitions.

## 5 Summary

It is clear that H3K4 methylation is intrinsically linked to CGIs in vertebrate chromatin. H3K4me3 deposition at CGIs appears to shape a chromatin environment around gene promoters that supports communication with the transcriptional machinery. However, the effects of perturbing the H3K4me3 system on gene expression are not straightforward and the mechanisms governing the role of CGI-associated H3K4me3 in the regulation of gene expression remain poorly understood. Due to the potentially bistable nature of CGI chromatin, we envisage that single cell technologies that capture the dynamic behaviours of gene expression will be required to enable further investigation into how H3K4me3 contributes to gene regulation.

## CrediT authorship contribution statement

**Amy L. Hughes:** Conceptualization, Writing - original draft, Writing - review & editing. **Jessica R. Kelley:** Conceptualization, Writing - original draft, Writing - review & editing. **Robert J. Klose:** Conceptualization, Writing - original draft, Writing - review & editing, Supervision.

## Declaration of competing interest

The authors declare that they have no known competing financial interests or personal relationships that could have appeared to influence the work reported in this paper.
